# Expression of OATP Family Members in Hormone-Related Cancers: Potential Markers of Progression

**DOI:** 10.1371/journal.pone.0020372

**Published:** 2011-05-19

**Authors:** Heather Pressler, Tristan M. Sissung, David Venzon, Douglas K. Price, William D. Figg

**Affiliations:** 1 Molecular Pharmacology Section, National Cancer Institute, Bethesda, Maryland, United States of America; 2 Clinical Pharmacology Program, Medical Oncology Branch, National Cancer Institute, Bethesda, Maryland, United States of America; 3 Biostatistics and Data Management Section, National Cancer Institute, Bethesda, Maryland, United States of America; 4 Johns Hopkins University, Baltimore, Maryland, United States of America; Roswell Park Cancer Institute, United States of America

## Abstract

The organic anion transporting polypeptide (OATP) family of transporters has been implicated in prostate cancer disease progression probably by transporting hormones or drugs. In this study, we aimed to elucidate the expression, frequency, and relevance of OATPs as a biomarker in hormone-dependent cancers. We completed a study examining *SLCO1B3*, *SLCO1B1* and *SLCO2B1* mRNA expression in 381 primary, independent patient samples representing 21 cancers and normal tissues. From a separate cohort, protein expression of OATP1B3 was examined in prostate, colon, and bladder tissue. Based on expression frequency, *SLCO2B1* was lower in liver cancer (P = 0.04) which also trended lower with decreasing differentiation (P = 0.004) and lower magnitude in pancreatic cancer (P = 0.05). *SLCO2B1* also had a higher frequency in thyroid cancer (67%) than normal (0%) and expression increased with stage (P = 0.04). *SLCO1B3* was expressed in 52% of cancerous prostate samples and increased *SLCO1B3* expression trended with higher Gleason score (P = 0.03). *SLCO1B3* expression was also higher in testicular cancer (P = 0.02). *SLCO1B1* expression was lower in liver cancer (P = 0.04) which trended lower with liver cancer grade (P = 0.0004) and higher with colon cancer grade (P = 0.05). Protein expression of OATP1B3 was examined in normal and cancerous prostate, colon, and bladder tissue samples from an independent cohort. The results were similar to the transcription data, but showed distinct localization. OATPs correlate to differentiation in certain hormone-dependent cancers, thus may be useful as biomarkers for assessing clinical treatment and stage of disease.

## Introduction

Organic anion-transporting polypeptides (OATPs; encoded by *SLCO* genes) are involved in hepatocyte influx mechanisms and are overexpressed in some cancers [1], [2], [3]. Known ligands of the OATP family include myriad endogenous substrates such as: steroids (i.e., estrogen sulfate, testosterone, and DHT), bile acids, and peptides [4]. OATP family members also influx a variety of pharmaceuticals, including (but not limited to): antihistamines, blood-glucose lowering drugs, statins, heart medications, and anticancer agents [4]. Therefore, OATPs are emerging as important transporters in the treatment of cancer from the standpoints of drug distribution and disease outcomes, and it is expected that many more endogenous and exogenous substrates will be identified by future studies.

Expression and genetic variation in OATPs have been associated with clinical outcomes in patients with prostate cancer. Both *SLCO1B3* and *SLCO2B1* have been related to a shorter time to androgen deprivation therapy failure and decreased overall survival in patients with hormone responsive prostate cancer, and castration resistant prostate cancer (CRPC) respectively [1], [5], [6]. Mechanistically, these associations might be attributed to genetic variation in androgen (or other steroid hormone) influx mechanisms that increase androgen scavenging during androgen deprivation therapy (ADT) [1]. Although OATP1B3 influx mechanisms are still unknown in colon cancer, there is sufficient evidence suggesting that OATP1B3 is overexpressed in a large proportion of colon tumors, and that this overexpression contributes to cell survival in the presence of oxalaplatin and camptothecin; the latter effect may be related to p53 expression [2].

OATPs have also been shown to contribute to systemic variation in anti-cancer drug treatments. For instance, OATP1B3 was initially identified as a high-affinity hepatocellular transporter of paclitaxel, and is also the most efficient liver uptake transporter of docetaxel [7], [8]. OATP1B1 and OATP1B3 also influx SN-38 [4], and this transport may have implications in the treatment of colon cancer [2]. However, the tumor influx mechanisms have yet to be explored for these drugs in relevant diseases, and this effort has been hindered by the lack of studies that have evaluated tumor expression of OATPs.

As OATPs have been implicated in both the etiology and treatment of prostate cancer and colon cancer, we undertook to explore whether or not these OATPs are also overexpressed in other tumor types and whether or not OATP expression could serve as a biomarker for tumor development or tumor aggressiveness in certain diseases.

## Materials and Methods

### Clinical samples

Samples were obtained from Origene (TissueScan Cancer Survey Panel III, Rockville, MD). Briefly, RNA was isolated from patients of mixed age, clinical diagnosis, and with various tumor stages. cDNA was created, normalized to actin B, checked for contamination and sensitivity (as determined by the manufacturer). The panel includes 384 samples encompassing 22 cancers and matched normal tissues. The numbers of samples corresponding to different tumor types and the number of samples derived from different tumor grades are reported in **Table S1**.

### Determination of mRNA expression

SLCO1B3 (Hs00251986_m1), SLCO2B1 (Hs00200670_m1), and SLCO1B1 (Hs00272374_m1) expression levels were analyzed using Taqman Gene Expression Assays (Applied Biosystems). Briefly, the primers were mixed with Taqman Master Mix (No Amp Erase) and distilled water then added to the samples. The plates were run in duplicate on Mx3005P QPCR System (Agilent Technologies) for 10 minutes 95°C then cycling for 42 cycles of 30 seconds for 95°C and 1 minute 60°C then FAM (ROX reference) reading after every cycle. Expression of each SLCO gene was normalized to actin by determining the cycle threshold (Ct) for each gene by the following formula: 2∧-(Ct_SLCO_-Ct_ActinB_)*10^5^ (i.e., the ΔCt). In cases where mRNA samples were below the LLOQ, the samples were noted to have *SLCO* mRNA levels at just below LLOQ (i.e. 42.1 cycles) and were normalized to actin as described above. This is considered to be a conservative adjustment as there were a large number of samples with undetectable *SLCO* mRNA levels that were likely to be significantly lower than the LLOQ.

### Determination of protein expression

Tissue section arrays for bladder, colon, and prostate were obtained from Pantomics (Richmond, CA). The prostate tumor tissue array includes benign prostatic hyperplasia and cancerous tissue; the normal and tissue samples were not matched per patient. Bladder and colon cancer arrays were also obtained from the above company and these included uninvolved and cancerous tissue. All tissues were obtained from surgical resection and fixed in 10% neutral buffered formalin for 24 hours. Slides were preparing by baking for 1 hour 60°C before removing the paraffin in xylene, then prepping for staining in 100% ethanol, 95% ethanol, 70% ethanol, and distilled water washes. One slide per set was stained with hematoxylin and eosin. The remaining slides were processed for immunoflourescence by placing in 95°C 10 mM sodium citrate buffer for 5 minutes then allowing to cool for 20 minutes. The slides were blocked for 30 minutes before primary antibody against OATP8 was applied 1∶100 (Progen) at 4°C overnight. Slides were washed in Tris-Buffered Saline Tween before applying the secondary FITC goat anti-mouse 1∶400 (Abcam) for 2 hours. Slides were washed again in TBST and mounted with VectaShield (Vector Labs) with DAPI. Images were taken on an Olympus BX51 microscope with UPlanFl 40x and 10x lenses. InSight Firewire camera and Spot version 4.5 imaging software were used to capture images. Adobe Photoshop CS3 version 10 was used for after-capture edits where all photos were processed the same.

### Statistical considerations

Comparisons between the frequency of *SLCO* or OATP expression in normal versus tumor tissues was conducted using Fisher's exact test. Comparisons were made between the magnitude of *SLCO* mRNA expression versus the corresponding normal tissue using the Wilcoxon rank sum test; these data are reported as the mean ΔCT (95% confidence interval; CI) and corresponding fold-change from normal tissue. The Cochran-Armitage trend test was employed to determine if the frequency of *SLCO* or OATP expression varied with tumor cell differentiation or stage, while the Jonckheere-Terpstra trend test was used to determine if the magnitude of *SLCO* mRNA expression varied with tumor cell differentiation or stage. Assessment of protein expression magnitude was not possible, although we did note if tumors expressed detectable levels of OATP1B3 or not. Only two-tailed *P*-values are reported, and given the exploratory nature of this study, *P*-values are reported as significant if P<0.05; however, it should be noted that conservative non-parametric and exact tests were performed in order to reduce the potential for false positives.

## Results

### Expression of SLCO mRNA in normal and tumor tissues

**Figure 1:**
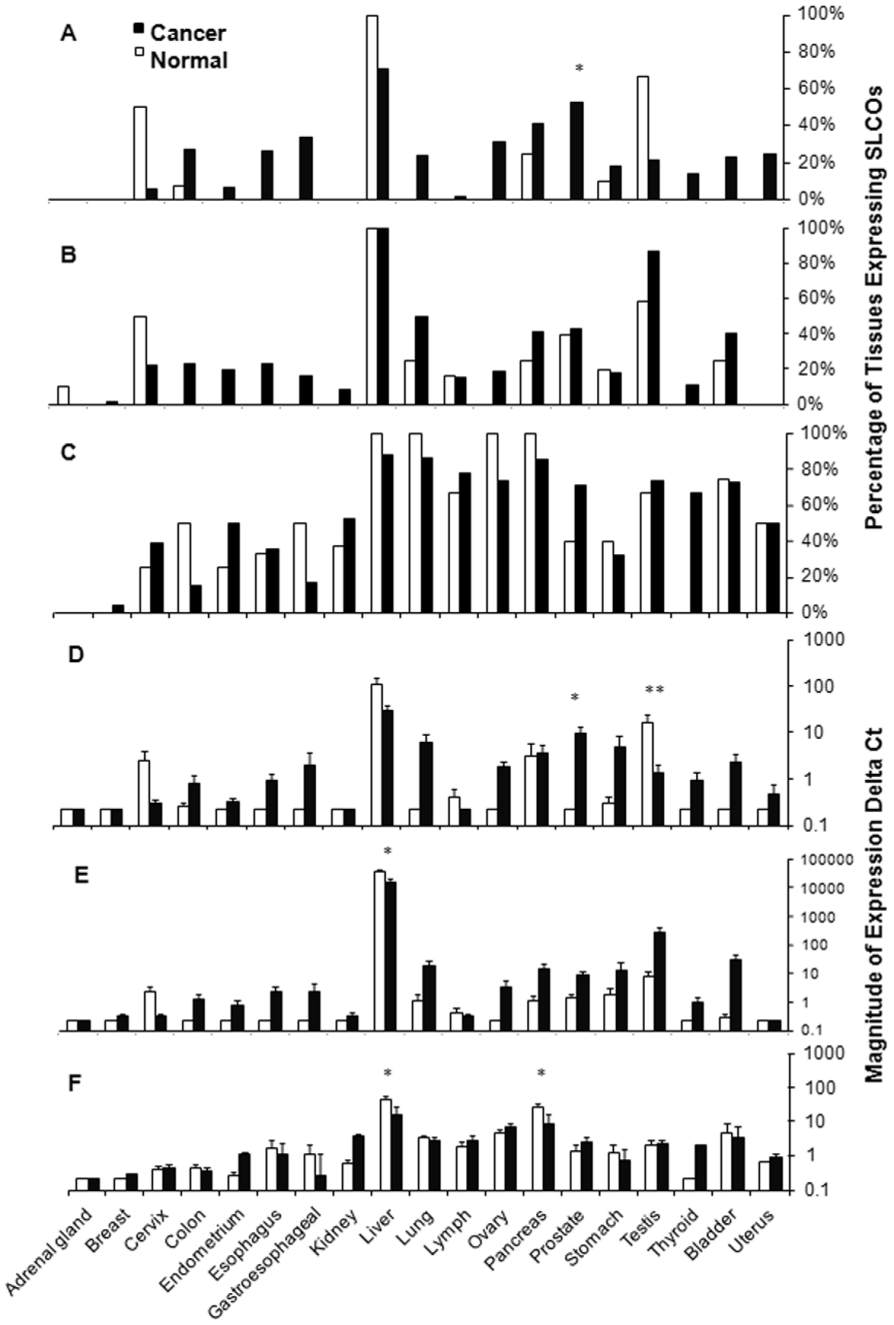
Expression profile of SLCOs in normal and neoplastic tissues. Data are first expressed as a percentage of tissues with mRNA expression of A) *SLCO1B3* B) *SLCO1B1*, and C) *SLCO2B1*, and then as the magnitude of normalized mRNA expression of D) *SLCO1B3*, E) *SLCO1B1*, and F) *SLCO2B1*. * - P<0.05, ** - P<0.01 for Fisher's Exact and Wilcoxon Rank Sum Tests respectively.

We first hypothesized that SLCO expression would be a biomarker for multiple different hormone-dependent tumor types. To this end, *SLCO* mRNA expression was determined in samples derived from normal human tissue and corresponding tumor tissue. Expression frequencies were determined for *SLCO1B3*, *SLCO1B1*, and *SLCO2B1*, and the percent of normal or tumor tissues expressing *SLCOs* are reported (see **Figure 1A–C** respectively). Prostate tumors expressed *SLCO1B3* much more frequently than normal prostate tissues (62% vs. 0%, *n* = 21 and *n* = 5 respectively; *P* = 0.04) and more frequently with increasing Gleason score (*P* = 0.03). In addition, *SLCO1B3* expression was less frequent in testicular tumors (21% vs. 67%, *n* = 19 and *n* = 6 respectively; *P* = 0.06). *SLCO1B1* was frequently expressed significantly higher with decreasing differentiation in colon tumors (*P* = 0.04). *SLCO2B1* was not expressed significantly different in cancer compared to normal tissue. In addition to the above observations, it should be noted that there were several cases where there was no *SLCO* expression in normal tissues, but expressed in cancerous samples. While not statistically significant due to low power, some tumor tissues have *SLCO* expression and should be evaluated more closely in future studies. These tumors included (for *SLCO1B3*); colon, endometrium, esophagus, gastroesophageal, kidney, ovary, and thyroid, (for *SLCO1B1*); cervix, endometrium, esophagus, gastroesophageal, lung, lymph, ovary, thyroid, bladder, and uterus (for *SLCO2B1*); colon, bladder (See **Table S1** for comprehensive data analysis).

As some tumor tissues had similar *SLCO* expression frequencies as normal tissues, we also ascertained if the magnitude of *SLCO* expression was different for *SLCO1B1*, *SLCO1B3*, and *SLCO2B1* (see **Figure 1D–F** respectively). Significant differences in expression levels (i.e. mean ΔCt_normal_ (95%CI) vs. ΔCt_tumor_(95%CI)) between normal and tumor tissue were observed for *SLCO1B3* in prostate cancer [0.23 (0.23 to 0.23) versus 104 (44 to 164); 45.7-fold increase; *P* = 0.03] and testicular cancer [174 (0.3 to 350) versus 14.0 (0.3 to 26); 12.8-fold decrease; *P* = 0.01]. Significant increases were seen for *SLCO1B1* in liver cancer [396000 (225000 to 567000) versus 161000 (41000 to 280000); 2.4 fold decrease; *P = *0.04]. *SLCO2B1* expression decreased significantly in pancreatic cancer [27.5 (9.5 to 45) versus 9.1 (5.5 to 12.8); 3.0 fold decrease; *P* = 0.05] and liver cancer [47.3 (16.1 to 78) versus 17.0 (4.9 to 29); 2.7 fold decrease; *P* = 0.04]. A more comprehensive analysis of expression differences is provided in **Table S1**.

### OATP expression versus tumor differentiation and stage

**Figure 2:**
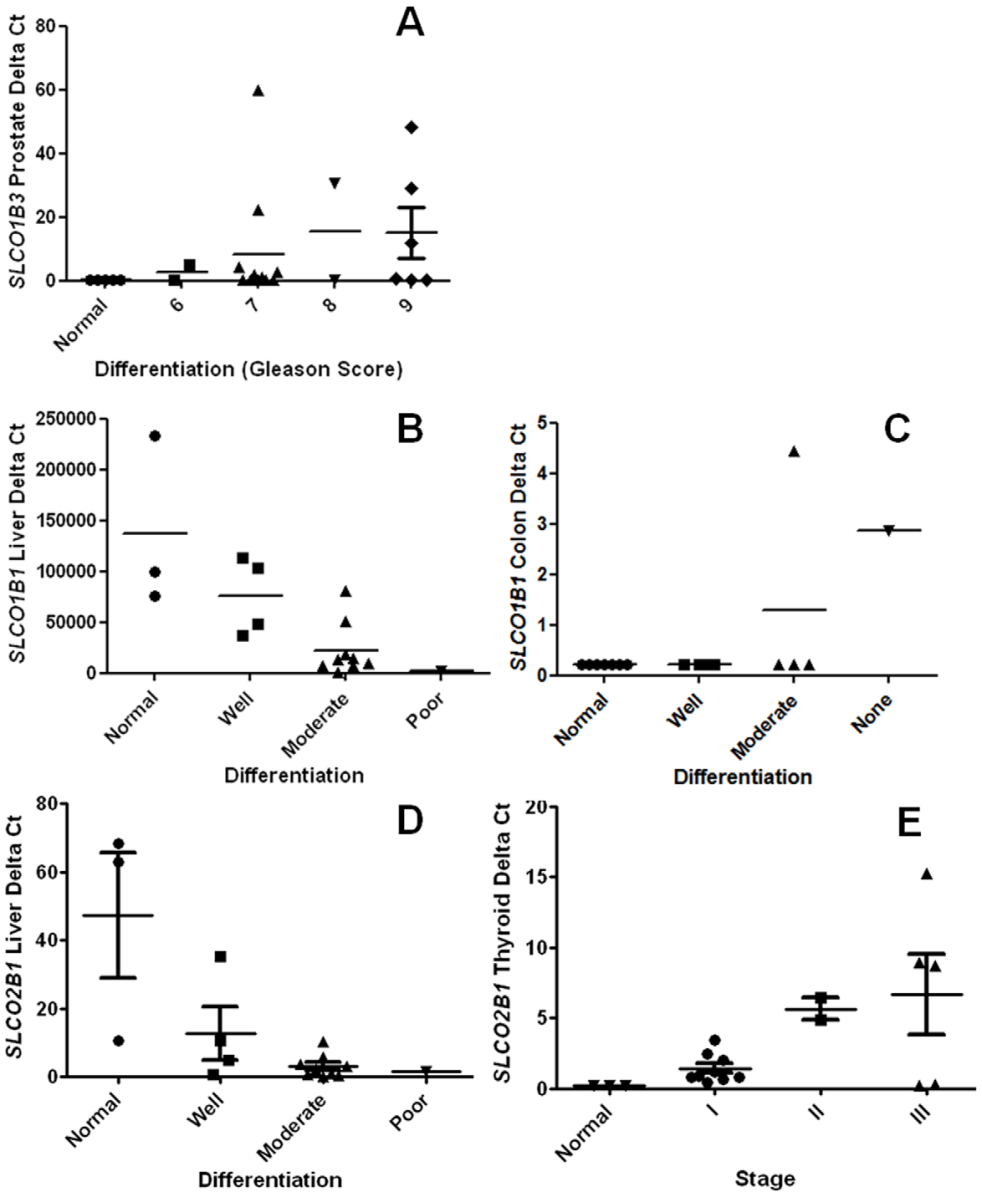
SLCO mRNA expression correlates to differentiation in cancer. *SLCO1B3* mRNA expression by differentiation in A) prostate P = 0.03. *SLCO1B1* expression in B) liver P = 0.0004, C) colon P = 0.05. *SLCO1B2* expression in D) liver P = 0.005. E) *SLCO2B1* expression by stage in thyroid P = 0.04. All were found as signifcant correlations by the Jonckheere-Terpstra trend test.

Since previous reports indicated that certain OATPs were involved in disease etiology and progression [1], [2], [5], we hypothesized that *SLCO* expression would be related to increasing tumor differentiation or stage. For prostate tumors, expression of *SLCO1B3* increased along with Gleason score up to 67-fold [mean ΔCT (95%CI)  = 0.23 (0.23 to 0.23), 2.6 (−4 to 9.2), 8.5 (−0.7 to 17.7), 15.5 (−13 to 44), 15.0 (2.4 to 27), for normal tissue, and Gleason  = 6, 7, 8, 9 respectively; *P* = 0.03; **Figure 2A**]. *SLCO1B1* expression was related to differentiation in liver cancer (395000 (225000 to 567000), 175000 (4300 to 310000), 47000 (11000 to 83000), and 3500 (N/A) for normal tissue, and well, moderately, and poor differentiated tumor tissue respectively; *P* = 0.0004, **Figure 2B**) up to a −74 fold difference. *SLCO1B1* expression was also related colon cancer (0.23 (0.23 to 0.23), 0.23 (0.23 to 0.23), 1.3 (−1.0 to 3.6), and 2.9 (N/A) for normal tissue, and well, moderately, and undifferentiated tumor tissue respectively; *P* = 0.05, **Figure 2C**) up to a 12.5 fold difference. *SLCO2B1* expression was also correlated negatively to differentiation in liver cancer (47 (4.4 to 82), 13 (−3.2 to 28), 3.1 (1.4 to 4.7), and 1.7 (N/A) for normal tissue and well, moderately, and poor differentiated tumor tissue respectively; *P* = 0.005) up to a 26-fold change (**Figure 2D**).

The expression and frequency data was also analyzed with respect to stage. *SLCO2B1* expression increased with stage up to a 29 fold-difference in thyroid cancer (P = 0.04). See **Figure 2E**. For normal and stage I, II, III, IVA the increase was 0.23 (0.23 to 0.23), 1.4 (0.6 to 2.1), 5.6 (−0.8 to 12), 6.7 (−0.4 to 14), 1.0 (N/A). Due to limited samples numbers for each stage no other relationships were statistically significant. However, other notable trends were *SLCO2B1* decreased expression by stage in pancreatic cancer, *SLCO1B3* increased frequency by stage in prostate cancer, and *SLCO1B1* decreased expression by stage in liver cancer. For the full data set see **Table S2**.

### OATP protein expression versus tumor differentiation

**Figure 3:**
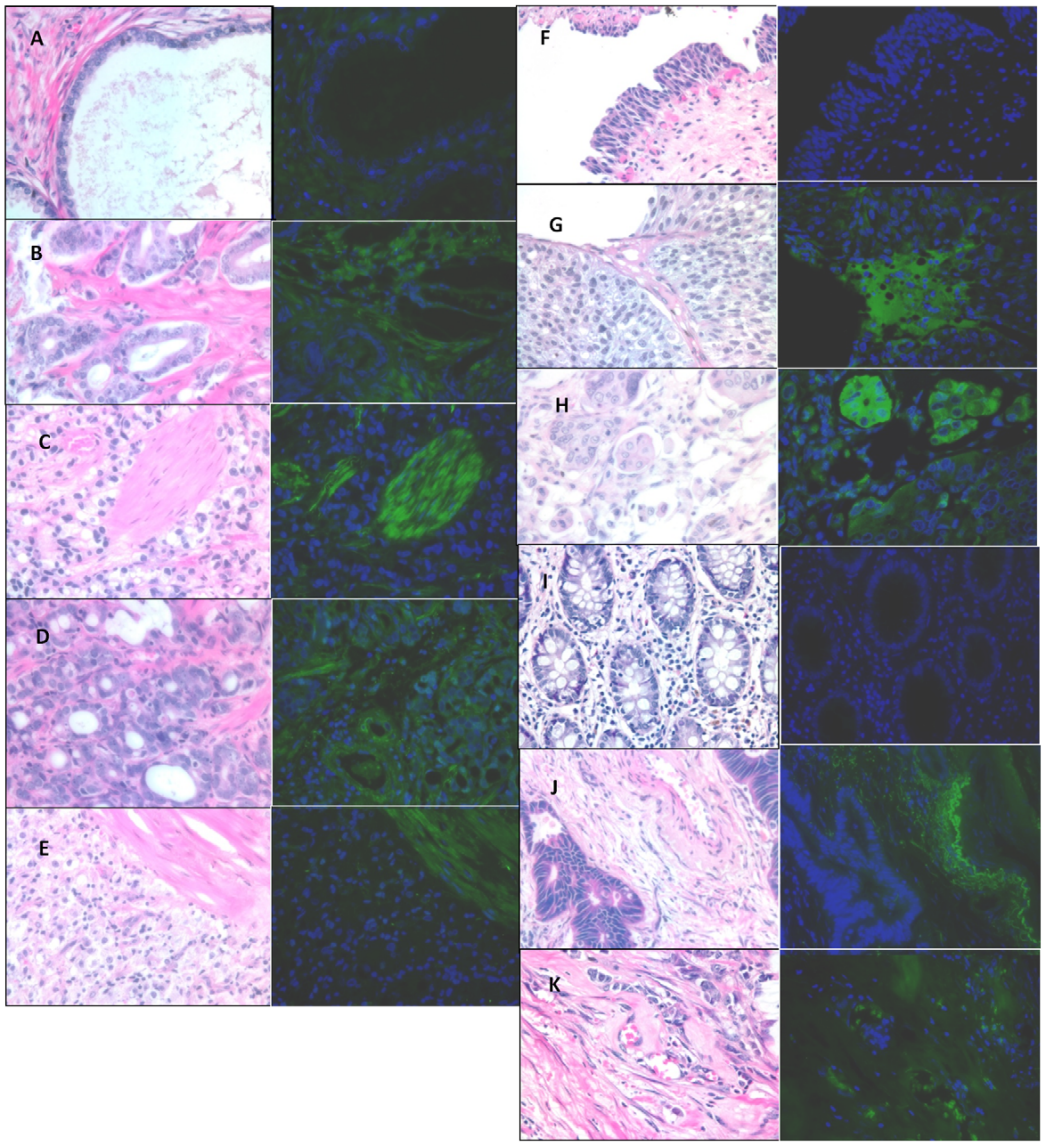
Tissue staining of OATP1B3 expression in cancer. Concurrent tissue sections stained with hematoxylin and eosin (left panel) and immunofluoresence (right panel) for OATP1B3. OATP1B3 is detected with FITC, in green, and nuclei are stained with DAPI, in blue. Prostate tissues comprising, BPH, and tumors of varying grade are reported as follows: A) benign prostatic hyperplasia, B) gleason 6, C) gleason 7, D) gleason 8, E) gleason 9. Bladder tissue sections are derived from F) normal bladder, G) grade II, H) grade III bladder tumors. Finally, colon tissue from I) normal colon, J) grade II (10x magnification), K) grade III colon tumors are disclosed. All photos were taken at 40x magnification unless otherwise noted.

To determine if OATP translation is similar to the quantitiative PCR, we conducted immunoflourescence on tissue samples from prostate, colon, and bladder cancer to detect OATP1B3. OATP2B1 was not chosen for protein validation because it is expressed both in normal and cancerous tissues. For prostate tumors, expression of OATP1B3 was primarily observed in prostate tumors and not normal tissues (*P = *0.001; **Figure 3A–E** **and** **Table 1**) and was highly expressed in the stroma. Moreover, the frequency of OATP1B3 expression varied significantly with Gleason score (*P* = 0.001; **Table 1**). Expression was also more frequent in colon cancer (*P = *0.06, **Figure 3F–H** and **Table 1**) and trended towards an association with a statistically significant higher incidence of expression in colon cancer by Cochran-Armitage test (*P = *0.02; **Table 1**). OATP1B3 expression was observed primarily in the vasculature in colon cancer and invasive, cancerous epithelial cells in bladder cancer. Consistent with the mRNA data, OATP1B3 expression was not associated with increasing histological grades of bladder cancer (*P* = 0.34; see **Table 1** and **Figure 3I–K**), but trended towards an association with bladder cancer grades despite a low number of samples (*P* = 0.07; see **Table 1**).

**Table 1:** OATP Expression in Immunoflourescent Tissue Sections.

Tissue	Differentiation	Express	Total	P value
Prostate				**0.001**
	BPH	7 *(26%)*	27	***0.001***
	Gleason 4	4 *(44%)*	9	
	Gleason 5	4 *(57%)*	7	
	Gleason 6	5 *(71%)*	7	
	Gleason 7	4 *(100%)*	4	
	Gleason 8	16 *(64%)*	25	
	Gleason 9	9 *(64%)*	14	
Colon				0.06
	Normal	7 *(21%)*	32	***0.02***
	II**–**I	3 *(38%)*	8	
	II	4 *(50%)*	8	
	III	6 *(50%)*	12	
Bladder				0.34
	Normal	5 *(21%)*	24	*0.07*
	II**–**I	1 *(17%)*	6	
	III**–**II	3 *(33%)*	9	
	III	4 *(50%)*	8	

P-value  =  Fischer's exact or *Cochran-Armitage trend test.*

## Discussion

*SLCO* (and OATP) overexpression has already been shown to be an important factor in colon and prostate cancer (1,2); moreover variation in OATP function [1] appears to be related to clinical outcome of endocrine therapy in prostate cancer [5] as well as overall survival of men CRPC [1]. Previous reports also demonstrated significant decreases in expression of *SLCOs* in hepatocellular carcinoma; this phenomenon is most likely associated with the reduction of metabolic function due to dedifferentiation in liver tumors derived from patient samples [9]. The present study confirms the above observations and demonstrates for the first time that *SLCO* expression variability is also associated with several other tumor types, including: colon cancer, liver cancer, pancreatic cancer, prostate cancer, testicular cancer, and thyroid cancer. Despite previously published reports, *SLCO1B3, SLCO2B1*, and *SLCO1B1* were not highly expressed in our breast cancer samples [10], [11].

We also demonstrated that *SLCO1B3* expression levels are significantly related to the Gleason score in prostate cancer. *SLCO1B1* expression levels were significantly related to the degree of differentiation in liver and colon cancers. *SLCO2B1* expression was also significantly related to liver and thyroid cancer. Immunoflourescent for OATP1B3 showed distinct localization in colon, bladder, and prostate cancers. The vasculature was stained for OATP1B3 only in high-grade colon cancer, cancerous epithelia in bladder cancer, and the stroma in prostate cancer. Thus OATP1B3 could be involved either directly or indirectly supporting carcinogensis depending on the cancer type. Previously mentioned studies suggest that variation in the expression of OATPs is likely involved in underlying disease etiology through the influx of endocrine factors.

OATPs influx several steroid hormones (i.e. DHEA, testosterone, DHT, and estrogen-sulfate) that underlie the etiology of several different diseases. For clarity, a table of selected OATP substrates is included (**Table 2**). Testosterone is a substrate for OATP1B3 and may explain its role in prostate[1], bladder [12], [13], and testicular [14] tumor development and progression. OATP1B3 and OATP1B1 also influx estrone-3-sulfate and thyroxine and these hormones play a major role in ovarian [15] and thyroid cancers [16] respectively. The OATP substrates could also inhibit cancer growth, thus be negatively selected against as the cancer progresses. For example, DHEA-S inhibits pancreatic cancer growth [17], [18], which might explain the observation that *SLCO2B1* expression is reduced in pancreatic cancer. In addition, the present data may also indicate a previously unknown role for the OATP-transported hormones in these cancers. Finally, OATPs transport a wide range of substances, thus there could also be unidentified or uncharacterized substrates that influence disease progression in the above-mentioned cancers.

**Table 2:** Select Substrates of OATPs[3], [21].

	Substrate
OATP1B1	
Anti-Cancer Drugs	ACU-154
	antamanide
	Bamet-R2
	Bamet-UD2
	demethyl phalloidin
	dihydromicrocystin-LR
	irinotecan
	ketoconazole
	methotrexate
	paclitaxel
	PKI-166
	SN-38
Hormones	estrone-3-sulfate
	estradiol-17B-glucuronide
	prostaglandin E2
	thyroxine (T4)
	triiodothyronine (T3)
OATP1B3	
Anti-Cancer Drugs	demethylphalloin
	dihydromicrocystin-LR
	docetaxel
	imatinib
	irinotecan
	methotrexate
	paclitaxel
	SN-38
Hormones	DHEAS
	testosterone
	estrone-3-sulfate
	estradiol-17B-glucuronide
	thyroxine (T4)
	triiodothyronine (T3)
OATP2B1	
Anti-Cancer Drugs	unknown
Hormones	DHEAS
	estrone-3-sulfate
	pregnenolone sulfate
	prostaglandin E2

Although it remains poorly explored, OATP expression may influence treatment success in certain cancer types (**Table 2**). OATP1B3 influxes docetaxel, paclitaxel, imatinib, irinotecan, SN-38, and methotrexate while OATP1B1 influxes ketoconazole, paclitaxel, and SN-38. OATP2B1 drug substrates remain poorly explored. Interestingly, these drugs are often very effective in treating metastatic diseases that develop as a result of the primary tumors that were evaluated herein. Docetaxel is approved for the treatment of CRPC and non-small cell lung cancer (NSCLC) and OATP1B3 is highly expressed in both prostate and lung tumors suggesting that the effectiveness of docetaxel treatment in these diseases may be, in part, due to sensitivity to docetaxel resulting from the underlying disease etiology. Using prostate tumors as an example, it appears that OATP1B3 is overexpressed during disease development in the primary tumor, facilitates the survival of metastatic prostate lesions during androgen deprivation therapy [1], [5], [19], and may be involved in the sensitivity of CRPC tumors towards docetaxel due to increased uptake. Moreover, *SLCO1B1* overexpression in prostate tumors may also explain the sensitivity of prostate cancer to ketoconazole. Similar relationships may be proposed for use of paclitaxel (in ovarian, lung, and esophageal tumors), imatinib (in gastroesophageal tumors), irinotecan and SN38 (in colon and lung tumors), and methotrexate (in lung tumors). Moreover, previous data have indicated that treatment- and disease-related *SLCO1B3* expression in liver result in differences in pharmacokinetic exposure to drugs [20]. We therefore propose the hypothesis that OATPs are involved in multidrug sensitivity through influx mechanisms and drugs targeting OATP influx may be more effective in certain diseases and treatment contexts based on OATP expression in the tumor and in the liver.

Limitations in this work include low positive tumors for several sample sets, so complex analysis on differentiation and expression could not be adequately assessed. In addition, little to no differentiation data was available for testis, thyroid, lymph, uterus, and gastroesophageal samples. As mentioned above, there are several substrates of OATPs that may explain its role in the identified cancers so further follow up studies should include clinical validation as well as functional studies related to substrate transport. Clinical follow up investigation for individual cancers is clearly warranted and should include examining expression in larger cohorts and confirming the effect of transported substrates on disease progression in thyroid, endometrial, lung, ovary, pancreas, testis, and bladder cancers.

This is the first study indicating that *SLCO1B1*, *SLCO1B1*, and *SLCO2B1* is significantly expressed (or expression is reduced) in a variety to tumors, including: colon cancer, liver cancer, pancreatic cancer, prostate cancer, testicular cancer, and thyroid cancer. We also propose that OATP expression may be a biomarker in prostate and colon cancers, and knowledge of tumor expression of OATPs could guide chemotherapy treatment. We conclude OATP expression may have implications on disease etiology and effectiveness of treatment, and should be studied further for its expression in cancer.

## Supporting Information

Table S1 **Tissues with detectable expression of SLCO1B1, SLCO1B3, and SLCO2B1.**
(XLS)

Table S2 **Expression of SLCO1B1, SLCO1B3, and SLCO2B1 by cancer stage.**
(XLS)
